# Vulnerability and non-adherence to treatment in cisgender women living with HIV/AIDS: a scoping review (2000–2024)

**DOI:** 10.1186/s13690-026-01852-z

**Published:** 2026-02-11

**Authors:** Denise Eliziana de Souza, Cleber Nascimento do Carmo, Simone Monteiro

**Affiliations:** 1 National School of Public Health Sergio Arouca, Oswaldo Cruz Foundation, Rio de Janeiro, Brazil; 2 Oswaldo Cruz Institute, Oswaldo Cruz Foundation, Rio de Janeiro, Brazil

**Keywords:** Vulnerability, Non-adherence, Cisgender women, HIV/AIDS

## Abstract

**Background:**

Cisgender women are disproportionately affected by HIV/AIDS due to structural gender inequalities and intersecting social determinants, which generate vulnerability contexts that act as barriers to treatment adherence.

**Methods:**

A scoping review to map studies examining vulnerability contexts influencing antiretroviral treatment non-adherence and discontinuation among cisgender women living with HIV/AIDS. Searches were performed in PubMed, SciELO, Scopus, Web of Science, and gray literature. A total of 78 studies published between 2000 and 2024 were included.

**Results:**

Findings were organized across three vulnerability dimensions individual, social, and programmatic. Stigma emerged as a transversal element permeating all levels. Barriers included mental health conditions, substance use, pregnancy and postpartum dynamics, socioeconomic inequalities, gender-based violence, and structural barriers in health systems. A conceptual model, grounded in the socio-ecological perspective and the vulnerability framework, is proposed to illustrate how these factors interact to affect adherence.

**Conclusion:**

Vulnerability contexts significantly shape women’s ability to maintain antiretroviral therapy. Addressing these challenges requires policies and interventions that integrate biomedical, social, and structural perspectives, while recognizing women’s autonomy and promoting equity in HIV care.

**Trial registration:**

This scoping review was conducted according to a previously published protocol, registered at Open Science Framework (https://osf.io/2ah3x/).

**Supplementary Information:**

The online version contains supplementary material available at 10.1186/s13690-026-01852-z.

**Table:** 

Text box 1. Contributions to the literature
- Maps evidence (2000–2024) on vulnerability contexts associated with antiretroviral treatment non-adherence and discontinuation among cisgender women living with HIV/AIDS.
- Shows that stigma intersects mental health symptoms, violence, and socioeconomic deprivation across individual, social, and health-system levels to undermine adherence.
- Highlights pregnancy and the postpartum period as a high-risk window for disengagement due to service transitions, caregiving demands, and disclosure-related pressures.
- Identifies policy and practice entry points for gender-responsive care, including stigma and violence-informed services, strengthened continuity across maternal and HIV/AIDS services, and social protection to reduce structural barriers.

## Introduction

Human Immunodeficiency Virus and Acquired Immunodeficiency Syndrome (HIV/AIDS) affects population groups in unequal ways. In 2024, an estimated 40.8 million people were living with HIV/AIDS worldwide. In the same year, approximately 1.3 million new infections were reported, and around 630,000 deaths occurred due to AIDS-related causes. Women and girls accounted for nearly 45% of all new infections globally, highlighting persistent gender-based inequalities in HIV vulnerability. In sub-Saharan Africa—a region marked by pronounced structural and social vulnerabilities—cisgender women and girls represented approximately 63% of new HIV infections, with a substantial concentration among women of reproductive age [1]. As an illustrative national context, data from Brazil indicate that 81.4% of reported HIV cases in 2024 occurred among individuals aged 15 to 49 years, alongside a growing proportion of diagnoses among women aged 50 years or older, increasing from 10.9% in 2014 to 17.0% in 2024 [2]. These trends underscore the need to consider age, gender, and context-specific vulnerability dynamics when examining antiretroviral treatment engagement among women living with HIV.

Gender inequalities intersect with social, racial-ethnic, generational, and regional disparities, heightening women’s vulnerability to HIV infection and creating barriers to sustained treatment adherence. These vulnerabilities are not restricted to individual behavior, but are shaped by structural and relational dynamics, such as unequal power relations, stigma, and limited access to resources. At the same time, women demonstrate autonomy in negotiating health care, disclosing their diagnosis, and mobilizing support networks, which highlights the complexity of adherence behaviors [3].

The concept of social determinants of health (SDH) provides a useful starting point for understanding these inequalities, encompassing individual, social, and structural conditions that affect health outcomes [4, 5]. Dahlgren and Whitehead [6] proposed that these determinants be arranged in layers, from individual characteristics to macro-structural conditions. Complementing this perspective, the socio-ecological model emphasizes the interactions between levels of influence—individual, relational, community, and institutional—highlighting their interdependence [7].

Applied to the HIV/AIDS context, theoretical approaches aim to identify how inequalities affect infection risks, access to care, as well as health services [8]. The concept of vulnerability in the health area emphasizes the interaction among these factors, taking into consideration the cultural, institutional, geographical, as well as informational components [9].

Building on these perspectives, the concept of vulnerability has been widely applied in the field of HIV/AIDS. Mann, Tarantola and colleagues [10, 11] advanced the notion that vulnerability goes beyond individual risk, incorporating social, economic, cultural, and institutional factors that shape exposure, prevention, and care. Later, Ayres and collaborators [12, 13] proposed a three-dimensional framework—individual, social, and programmatic vulnerabilities—to analyze how these dimensions interact and condition health outcomes. This perspective emphasizes that health care outcomes are not merely the consequence of individual choices but rather emerge from a complex web of interactions among individuals, societal structures, and institutional frameworks. Such an approach offers a conceptual basis for comprehending the multifactorial realities that shape the lived experiences of people living with HIV/AIDS (PLWHA). This framework is particularly useful for examining adherence to antiretroviral therapy (ART), as it situates women’s experiences within broader social and programmatic contexts.

In this review, we operationalize the vulnerability framework by charting and synthesizing evidence into three interrelated dimensions: individual vulnerability (e.g., mental health symptoms, substance use, treatment beliefs, caregiving burden), social vulnerability (e.g., stigma, violence, poverty, gender norms, partner dynamics, social support), and programmatic vulnerability (e.g., service accessibility, continuity of care, communication with providers, health system fragmentation) [12, 13]. This structure supports a socio-ecological interpretation of adherence, emphasizing that treatment engagement is shaped by interactions between personal circumstances, social relations, and health system arrangements rather than individual choices alone [7].

Aligned with this theoretical basis, the present review investigates how vulnerability contexts affect antiretroviral treatment adherence among women living with HIV/AIDS (WLWHA). In order to deepen the understanding of factors associated with non-adherence, this article sought to map the existing academic literature on the subject, identifying determinants of non-adherence; here understood as the failure or inconsistency in following prescribed therapeutic regimens, which may compromise the effectiveness of continuous antiretroviral therapy and related clinical monitoring indicators [14].

## Methods

This study consists of a scoping review of the academic literature addressing vulnerability contexts that hinder HIV/AIDS treatment adherence among cisgender women. The review was conducted in accordance with the Preferred Reporting Items for Systematic Reviews and Meta-Analyses—Extension for Scoping Reviews (PRISMA-ScR) guidelines [15] and was guided by the methodological framework proposed by the Joanna Briggs Institute (JBI) [16]. The process involved six main key steps: (1) identification of the research question; (2) identification of relevant studies aligned with the objectives of the review; (3) selection of studies based on predefined inclusion and exclusion criteria; (4) data charting; (5) synthesis of findings through thematic analysis; and (6) presentation of results, highlighting implications for policy, clinical practice, and future research. The review protocol was registered on the Open Science Framework platform (OSF) [17].

### Searches

The research question, constructed using the population-concept-context (PCC) strategy, is: How do various vulnerability contexts impact the failure to adhere to and abandonment of antiretroviral treatment by cisgender women living with HIV/AIDS? The review focused on three analytical dimensions of vulnerability—individual, social, and programmatic—based on the framework proposed by Ayres and collaborators [12, 13]. Accordingly, studies addressing factors related to non-adherence among WLWHA were included, encompassing conference abstracts, guidelines, books, editorials, primary studies employing qualitative, quantitative, or mixed methods, reviews, theses, and dissertations. No restrictions were imposed regarding language, publication dates, or locations.

The search was conducted between October and December 2024 across health-related databases, including the National Library of Medicine (PubMed), Scientific Electronic Library Online (SciELO), Scopus, and Web of Science. To include gray literature, searches were performed in the Digital Library of Theses and Dissertations (DLTD). Descriptors, including Medical Subject Headings (MeSH) terms, were: "cisgender women," "women," "female," "non-adherence," "treatment failure," "abandonment," "HIV," "Acquired Immunodeficiency Syndrome," "antiretroviral therapy," "ART," "antiretroviral treatment," and their Portuguese equivalents, combined using Boolean operators "OR" and "AND." Gray literature was searched in the DLTD using the same PCC logic and translated keywords aligned with the database strategy. Records retrieved from DLTD were exported and imported into Rayyan alongside bibliographic database results for deduplication and screening. Titles/abstracts were screened independently by two reviewers, followed by full-text screening using the same eligibility criteria applied to peer-reviewed studies. When necessary, additional document information (e.g., thesis institutional repository page) was consulted to confirm eligibility.

### Study inclusion and exclusion criteria

The study included peer-reviewed articles and gray literature that addressed WLWHA; reported on treatment adherence, discontinuation, or interruption; analyzed factors associated with individual, social, or programmatic vulnerabilities. Exclusion criteria were studies focusing exclusively on male or transgender populations; articles not addressing adherence or treatment interruption.

### Potential effect modifiers and reasons for heterogeneity

Not applicable to this scoping review, which aimed to map evidence rather than assess comparative effects or sources of statistical heterogeneity.

### Study quality assessment

Not performed, as this is a scoping review conducted in accordance with PRISMA-ScR guidance.

### Data extraction strategy

Studies were managed using the Rayyan Qatar Computing Research Institute platform [18] for duplicate removal and screening processes. Two reviewers independently screened titles and abstracts, followed by full-text reading of potentially eligible studies. Disagreements were resolved by consulting a third reviewer. The review team had full-text proficiency in Portuguese, English, and Spanish. For studies in other languages (e.g., French, Italian, Romanian), titles/abstracts were screened using available translations, and full texts were assessed with translation support when required, with key excerpts cross-checked by the reviewers to minimize misinterpretation. Subsequently, full-text readings were conducted for analysis and content summarization using a data extraction spreadsheet containing information on title, author, publication year, journal, country of study, objectives, population, methodology, main results, and final considerations.

### Data synthesis and presentation

Extracted data were analyzed using qualitative content analysis. Studies were organized according to the vulnerability framework proposed by Ayres and collaborators [12, 13]. Recurring themes were identified, and findings were synthesized narratively. Theoretical concepts of vulnerability and the socio-ecological perspective guide the interpretation of results. Finally, results were compiled through thematic construction based on elements influencing the research question's response.

## Results

### Review statistics

**Fig. 1:**
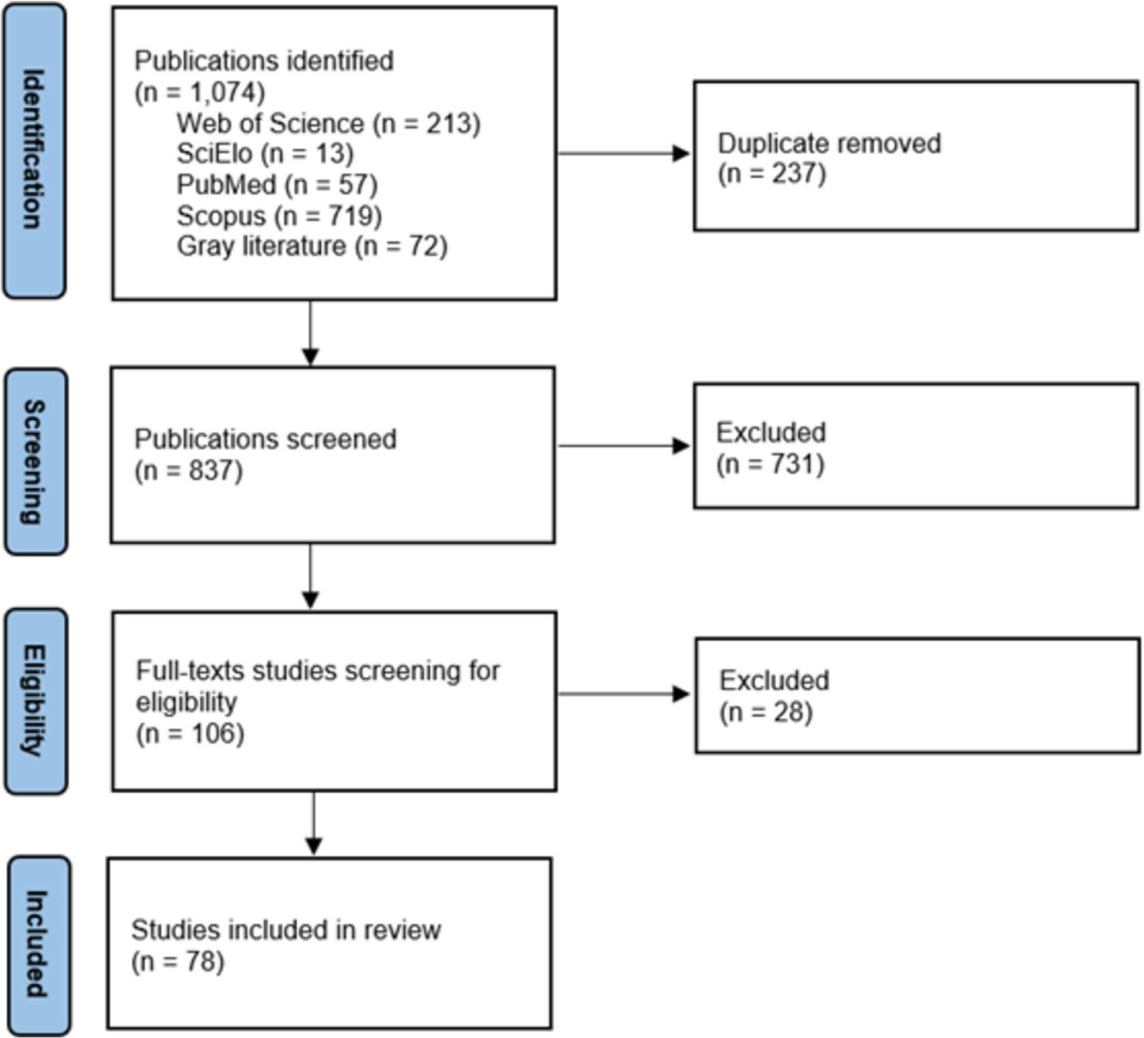
PRISMA-ScR flowchart results for a scoping review on vulnerability and non-adherence to treatment in cisgender women living with HIV/AIDS (2000–2024). Source: elaboration constructed by the authors. Note: Searches conducted October-December 2024 using Rayyan. PRISMA-ScR: Preferred Reporting Items for Systematic Reviews and Meta-Analyses – Scoping Review Extension

From 1,074 initial studies, 213 were identified in Web of Science, 13 in SciELO, 57 in PubMed, 719 in Scopus, and 72 in gray literature. After removing 237 duplicates, 837 studies remained. Of these, 731 were excluded after title and abstract screening for not meeting the review's objectives, leaving 106 studies for full-text reading and analysis, and 78 were included in this study (Fig. 1).

Among the 106 full-text reports assessed, 28 were excluded for reasons including: lack of women-specific results (e.g., mixed-gender samples without stratified analyses), absence of ART adherence/non-adherence outcomes, focus on prevention rather than treatment engagement, ineligible population (not cisgender women living with HIV), or insufficient information to determine adherence-related findings.

### Study quality assessment

Not applicable. No formal quality appraisal was conducted, consistent with the objectives of this scoping review.

### Quantitative synthesis/meta-analysis

Not applicable. No quantitative synthesis or meta-analysis was planned or conducted.

### Evidence of effectiveness

Not assessed, as this review aimed to map vulnerability contexts rather than evaluate intervention effectiveness.

### Study characteristics

The selected studies were published between 2000 and 2024 in Portuguese, English, Spanish, French, Italian, and Romanian. Included materials comprised peer-reviewed journal articles (85.9%), gray literature such as theses and dissertations (12.8%), and conference presentations (1.3%). The majority originated from the Americas (53.8%), followed by Africa (37.2%), Europe (7.7%), Asia (2.5%), and one multi-country study (1.3%) involving Argentina, Botswana, Brazil, China, the United States, Haiti, Peru, and Thailand. Quantitative studies predominated (64.0%), with qualitative approaches (18.0%), reviews and evidence syntheses (11.5%), and mixed-methods studies (6.4%).

Table 1 presents general characteristics of the studies, including author, year, population scope, and country coverage.

**Table 1:** Study characteristics (author, year, population, and country) extracted for a scoping review on vulnerability and non-adherence to treatment in cisgender women living with HIV/AIDS (2000–2024)

Authors	Year	Population	Country
Johnston et al	2000	WLWHA	United States
Sankar et al	2002	WLWHA	United States
Mellins et al	2002	WLWHA—mothers from ethnic minorities	United States
Murphy et al	2002	WLWHA—symptomatic or AIDS-diagnosed	United States
Wood et al	2004	WLWHA—mothers	United States
Schiopescu et al	2005	PLWHA	United States
Locher et al	2007	WLWHA—black women	United States
Mellins et al	2008	WLWHA—pregnant and postpartum	United States
Ubbiali et al	2008	PLWHA	Italy
Costa et al	2009	WLWHA	Brazil
Plankey et al	2009	WLWHA	United States
Psaros et al	2009	WLWHA—with depression	United States
Applebaum et al	2009	PLWHA	United States
Puskas et al	2011	PLWHA	United States
Felix et al	2012	WLWHA	Brazil
Christie et al	2012	WLWHA—pregnant women, exposed children, adolescents	Jamaica
Bonolo et al	2013	PLWHA	Brazil
Vitalis	2013	WLWHA	England
Phillips et al	2014	WLWHA—pregnant women	South Africa
Remien et al	2014	PLWHA	United States
Njuki et al	2014	WLWHA	Kenya
Padoin et al	2015	WLWHA	Brazil
Fagbami et al	2015	WLWHA	United States
Willie et al	2016	WLWHA—history of childhood sexual abuse	United States
Gameiro	2016	WLWHA—pregnant women	Brazil
Santa Helena	2016	WLWHA	Brazil
Phillips et al	2016	WLWHA—pregnant women	South Africa
Hatcher et al	2016	WLWHA—pregnant and postpartum	South Africa
Currier et al	2017	WLWHA—postpartum	Argentina, Botswana, Brazil, China, Haiti, Peru, Thailand, United States
Li et al	2017	PLWHA	United States
Lobato	2017	WLWHA—pregnant women	Brazil
Acheampong et al	2017	WLWHA—breastfeeding mothers	Ghana
Adeniyi et al	2018	WLWHA—postpartum	South Africa
Langwenya et al	2018	WLWHA	South Africa
Sikkema et al	2018	WLWHA	South Africa
Kadima et al	2018	WLWHA—pregnant women	Lesotho
Williams et al	2018	WLWHA—pregnant women	United States
Musoke et al	2018	Male partners of pregnant HIV-positive and negative women	Kenya
Omonaiye et al	2018	WLWHA—pregnant women	Sub-Saharan Africa
Ramlagan et al	2019	WLWHA	South Africa
Geter et al	2019	WLWHA	United States
Dlamini et al	2019	WLWHA—postpartum	Eswatini
Namale et al	2019	WLWHA	Uganda
Braga	2019	PLWHA	Mozambique
Coan	2019	WLWHA—pregnant women	Brazil
Conroy et al	2019	Serodiscordant and seroconcordant couples	Malawi
Tebeu et al	2019	WLWHA—pregnant women	Cameroon
Kisigo et al	2020	WLWHA—postpartum	Tanzania
Gouvea	2020	WLWHA	Brazil
Allam et al	2020	WLWHA—sex workers	India
Cutimanco-Pacheco et al	2020	WLWHA—pregnant women	Peru
Aduloju et al	2020	WLWHA—pregnant women	Nigeria
Marcu et al	2020	WLWHA—pregnant women	Romania
Garriga et al	2020	PLWHA	Spain
Medeiros et al	2021	WLWHA—pregnant and postpartum	Brazil
Candido et al	2021	WLWHA	Brazil
Peña et al	2021	WLWHA	Spain
Mukose et al	2021	WLWHA—pregnant women	Uganda
Cabral et al	2021	WLWHA	Brazil
Lorenzetti et al	2021	WLWHA—pregnant and postpartum	Malawi
Dada et al	2021	WLWHA—pregnant and postpartum	Nigeria
São Pedro	2022	WLWHA—pregnant women	Brazil
Myer et al	2022	WLWHA—postpartum	South Africa
Hatcher et al	2022	WLWHA	South Africa
Marinaro et al	2022	WLWHA	Italy
Argolo Júnior	2022	WLWHA—elderly women	Brazil
Minja et al	2022	WLWHA—pregnant women with depression	Tanzania
Barros	2023	WLWHA	Brazil
Pellowski et al	2023	WLWHA—pregnant and postpartum	South Africa
Palar et al	2023	WLWHA	United States
Wang et al	2023	WLWHA—sex workers	Dominican Republic
Martins et al	2023	PLWHA	Brazil
Batitucci	2023	WLWHA—pregnant women	Brazil
Stedile	2024	WLWHA—pregnant women	Brazil
Tusabe et al	2024	WLWHA—refugee pregnant women	Uganda
Tizianel et al	2024	PLWHA	Brazil
Heydari et al	2024	WLWHA	Iran
Zeleke et al	2024	WLWHA	Ethiopia

Data sources: Scopus, PubMed, SciELO, Web of Science, and Digital Library of Theses and Dissertations

*WLWHA* Women living with HIV/AIDS, *PLWHA* People living with HIV/AIDS

A significant proportion focused exclusively on WLWHA (84.6%); among these, 48.5% centered on pregnancy and/or postpartum contexts. Additionally, studies analyzing PLWHA by gender highlighted differences associated with increased vulnerability among women to HIV/AIDS.

### Vulnerability contexts of cisgender Women to HIV/AIDS

Analyzing factors related to non-adherence among WLWHA through vulnerability dimensions reveals interconnections among factors, emphasizing the importance of this analytical perspective in understanding and promoting treatment adherence.

Based on the vulnerability framework proposed by Ayres and collaborators [12, 13], Table 2 organizes the identified factors into individual, social, and programmatic dimensions.

**Table 2:** Thematic synthesis of individual, social, and programmatic vulnerabilities associated with non-adherence, extracted for a scoping review on vulnerability and non-adherence to treatment in cisgender women living with HIV/AIDS (2000–2024)

Dimension	Aspects
Individual	Age [28; 35; 42–62]; education level[47–48; 57–60]; pregnancy/postpartum [19; 42; 57; 58; 61; 63–69]; mental health [19–27]; alcohol and drug use [28–34]; attitudes and beliefs [26; 35–46]; time since diagnosis [28; 35; 42]; race/skin color [44; 56; 68; 78]; mode of transmission [70; 71]; body image perception [39; 59]; clinical status [63; 72; 76]
Social	Socioeconomic status [43; 79; 82]; food insecurity [46; 81]; stigma perception [47; 79–81]; social and family support [79; 80]; marital/relationship status [21; 54]; migrant/refugee status [83]; sex work [44; 73]; incarceration [83]; partner involvement [73; 80]; disclosure of HIV status [28; 66]; context of violence [21; 22; 35; 84]
Programmatic	Distance to healthcare services [24; 40; 85]; availability of ART [69; 78]; availability of healthcare staff [41; 50]; therapeutic regimen [79; 86; 87]; vertical transmission prevention protocol [55; 75]; provider-patient relationship [24; 80]; timing of ART initiation [72; 88]; institutional/governmental policies [32; 58; 66]

Data sources: Scopus, PubMed, SciELO, Web of Science, and Digital Library of Theses and Dissertations

*ART* Antiretroviral

### Individual vulnerability

Individual vulnerability encompasses a complex interplay of psychosocial, behavioral, and clinical determinants that significantly affect adherence to therapeutic regimens. Mental health disorders—particularly anxiety, depression, bipolar affective disorder (BAD), and post-traumatic stress disorder (PTSD) [19–27], constitute critical barriers to consistent treatment engagement. These conditions often impair cognitive functioning, emotional regulation and motivation, thereby undermining the patient’s ability to comply with prescribed interventions. Additionally, the consumption of both legal and illicit psychoactive substances has been consistently identified as a major impediment to adherence, further exacerbating treatment discontinuity and compromising health outcomes [28–34].

Negative or unfavorable attitudes and beliefs regarding antiretroviral therapy have also been identified as vulnerability factors [26, 35–42]. These factors influence behaviors that increase exposure to risks, such as non-adherence to preventive and self-care practices [43–46].

Younger women and those with a shorter time since diagnosis appear more prone to non-adherent behaviors [28, 35, 42–60]. Among older women, adherence behavior was generally satisfactory, although ignorance and fear were reported as dominant initial reactions to the diagnosis [61]. Another relevant aspect involves the experience of menopause; women experiencing symptoms are more likely to exhibit non-adherence compared to asymptomatic women [62].

Initiation of ART during later stages of pregnancy—possibly reflecting a late diagnosis—was associated with poorer adherence indicators [19, 61, 63–68]. The postpartum period is also significant: the literature indicates that better adherence during pregnancy is often driven by fear of vertical transmission of HIV to the baby. However, adherence behaviors typically decline after delivery [42, 57, 58]. These dynamics reflect social and structural expectations placed on women as caregivers, often prioritizing the well-being of others, particularly children, over their own health, especially in the context of motherhood [69].

Women infected through vertical transmission tend to demonstrate greater adherence compared to those infected via sexual transmission, possibly due to greater risk awareness and the lifelong internalization of self-care practices [70]. Furthermore, women diagnosed for longer periods are generally more adapted to their treatment routines, which facilitates adherence [65, 71]. Nevertheless, one study found that women diagnosed before 2005 were at higher risk of treatment interruption, possibly due to challenges related to the longitudinal nature of care [72].

Perceptions of antiretroviral therapy side effects can hinder treatment continuity and increase the risk of discontinuation [32, 53–55, 58, 59, 73–77]. Additional clinical factors, such as low CD4 + lymphocyte counts, late ART initiation, drug interactions from concurrent treatments, and the presence of comorbidities, complicate adherence due to the increased complexity of the therapeutic regimen and uncertainty about its effectiveness [63, 72, 76]. Body image concerns, particularly regarding fat redistribution or accumulation, also serve as predictors of non-adherence, adversely affecting women's attitudes toward their treatment [39, 59].

Lower educational attainment is associated with a reduced capacity to comprehend the importance of continuous treatment and the consequences of HIV infection, which may hinder adherence [47–49, 52, 53]. Women with lower education levels consistently demonstrate lower adherence rates than men, indicating greater vulnerability [54, 60].

Skin color and race also play a critical role: black women (including those identified as black or brown) and other ethnic minorities frequently experience worse adherence outcomes due to structural racism, discrimination, and barriers in accessing healthcare [44, 56, 68, 78]. Findings on race/ethnicity and adherence were reported predominantly in studies from the United States, where race/ethnicity variables were more consistently collected and analyzed; evidence from other regions was more limited and less comparable, which constrains cross-context generalizability.

Fear, related both to disease progression and to potential rejection, negatively affects adherence by creating psychological and social barriers [26, 35, 79, 80]. In this context, stigma is a particularly significant obstacle to treatment adherence. It is commonly linked to fears of discrimination and social exclusion, especially in communities where HIV is still surrounded by taboos [64, 66, 68].

These factors are often interwoven with other aspects of vulnerability, particularly those that fall within the social and programmatic dimensions, discussed in the following sections.

### Social vulnerability

Social vulnerability refers to economic and social factors that hinder adherence to treatment. Within this context, stigma is prominently cited in literature as a critical factor, significantly affecting the emotional well-being of WLWHA, with direct consequences for their treatment adherence. Experiences of stigmatization often lead to feelings of shame, social isolation, and lack of support, all of which jeopardize treatment continuity and intensify emotional and social vulnerability [47, 81].

Women who face stigma, whether in family settings, social environments, or within healthcare systems, tend to experience greater difficulty maintaining therapeutic adherence due to fear of judgment or rejection [79, 80]. As a result, many choose to conceal their HIV status, which limits social support and engagement with treatment networks [28, 66]. Marital or relational status also plays a significant role: women who maintain relationships with partners to whom they have not disclosed their HIV status or who do not participate in their treatment process are more likely to encounter difficulties with adherence [21, 54]. These factors together may influence women’s perceptions of the illness as well as the treatment, modulating adherence behaviors [44, 72].

Partner involvement may indeed offer both emotional and practical support, constituting a protective factor. Socio-familial support networks can help women cope with the challenges of living with HIV and undergoing antiretroviral therapy [73, 80].

Studies have also identified that socioeconomic aspects are decisive in shaping adherence behaviors and other health outcomes among WLWHA. Women with low income and educational levels often face significant access barriers to treatment [43, 79, 82]. In this regard, unemployment and food insecurity are particularly detrimental, contributing to worsened physical and mental health conditions and posing substantial obstacles to adherence [46, 81].

Contextual factors such as migrant or refugee status can further complicate access to treatment due to lack of healthcare access, language barriers, and uncertainties related to relocation [83]. Similarly, WLWHA who engage in sex work face multiple layers of vulnerability—including stigma, discrimination, and limited healthcare access—which negatively affect adherence [44, 73]. Notably, women in conflict with the law or experiencing incarceration exhibit the poorest levels of adherence [83], highlighting the impact of structural barriers on healthcare access.

Furthermore, the context of violence must be underscored as a compounding factor of vulnerability, intrinsically tied to gender-based inequality. According to the literature, women who experience violence may lack the resources, both emotional and practical, to seek and maintain treatment, particularly due to trauma [22]. Research on intimate partner violence (IPV) and studies involving WLWHA who experienced childhood abuse reveal that such contexts of violence significantly compromise adherence, including among pregnant women. These situations are often linked to poor clinical outcomes and mental health issues [21, 22, 35, 84, 85].

As demonstrated, individual and social factors, when interrelated with programmatic factors described in the next section, highlight the complex challenges that women living with HIV must navigate to maintain consistent treatment adherence.

### Programmatic vulnerability

Programmatic vulnerability refers to issues related to public policies and access to healthcare services. Public policies are expected to promote equity between individuals and health services, ensuring democratic access to healthcare resources. Programmatic factors include barriers to treatment access such as the complexity of therapeutic regimens, as well as unfavorable institutional attitudes and protocols that may discourage treatment continuity.

Structural barriers represent a fundamental challenge to HIV treatment adherence, especially within contexts of social and economic inequality. WLWHA in situations of vulnerability frequently encounter difficulties in initiating and maintaining adequate treatment due to distance from healthcare services or transportation challenges [24, 40, 41, 50, 69, 78, 86]. Moreover, the stigma associated with HIV may in fact discourage women from seeking medical services for fear of discrimination [43, 63].

Beyond concerns about side effects, the complexity of antiretroviral regimens may pose adherence barriers for WLWHA who struggle with comprehension, organization, and lack of psychological or social support [79, 87, 88]. In this regard, in addition to social support, a strong bond between the patient and healthcare providers may foster treatment engagement. Trust in healthcare professionals facilitates adherence, especially when women feel welcomed and understood. Studies highlight that positive interpersonal relationships with healthcare providers are among the most decisive factors for treatment success, whereas lack of rapport may lead to treatment abandonment [24, 80].

The HIV vertical transmission prevention protocol may also act as a barrier to adherence. Beyond subjective aspects, the demands and routines required by this protocol may complicate adherence. While pregnancy is often associated with improved adherence among WLWHA, the complexity and rigor of the protocol, along with the availability and workflow of healthcare services, may become obstacles. Structural barriers hinder both access and follow-up for pregnant women, compounding vulnerabilities related to pregnancy and the postpartum period [72, 85].

The timing of ART initiation is also a critical factor from a programmatic perspective. Starting treatment as soon as possible after diagnosis is essential to reduce viral load, prevent disease progression, and reduce transmission. Several studies indicate that delayed ART initiation, especially among young or pregnant women, increases the risk of complications and vertical transmission [58, 75]. Delays may stem from fear of diagnosis, late awareness, or difficulties accessing and connecting with healthcare services. Moreover, such delays are linked to institutional shortcomings, limited availability of reference services, and pervasive stigma.

The lack of effective public policies and inconsistent implementation of prevention and treatment programs also obstruct access to medication and care for many women [32]. Policies such as free medication distribution, accessible healthcare facilities, and awareness campaigns are essential to ensure that all women have equal opportunities for treatment. Furthermore, effective policies may help reduce access barriers and ensure that treatment is initiated and maintained appropriately [58, 66].

### Pregnancy and postpartum-specific vulnerabilities

Nearly half of the included studies focused on pregnancy and/or postpartum contexts. In this subset, programmatic vulnerabilities were often centered on transitions across services (e.g., antenatal care, delivery, postpartum follow-up) and disruptions in continuity of ART and monitoring. Social vulnerabilities were frequently mediated by disclosure concerns, partner dynamics, and stigma related to motherhood and vertical transmission. Individual-level factors included emotional distress, treatment fatigue, and competing caregiving demands. These patterns suggest that pregnancy/postpartum periods may concentrate intersecting vulnerabilities and represent a critical window for differentiated support to sustain ART engagement.

### Vulnerability and non-adherence in cisgender women

Based on the reviewed studies, a hypothetico-deductive model [89] is proposed to represent the relationships between individual, social, and programmatic dimensions of vulnerability among cisgender women living with HIV/AIDS. These dimensions are conceived as existing across proximal, intermediate, and distal levels, in accordance with the Social Determinants of Health framework [6] and the socio-ecological model [7]. This model maps the components of each vulnerability dimension as outlined in Table 2. Non-adherence is defined here as the failure or inconsistency in following prescribed treatment guidelines, negatively impacting adherence indicators [14].

**Fig. 2:**
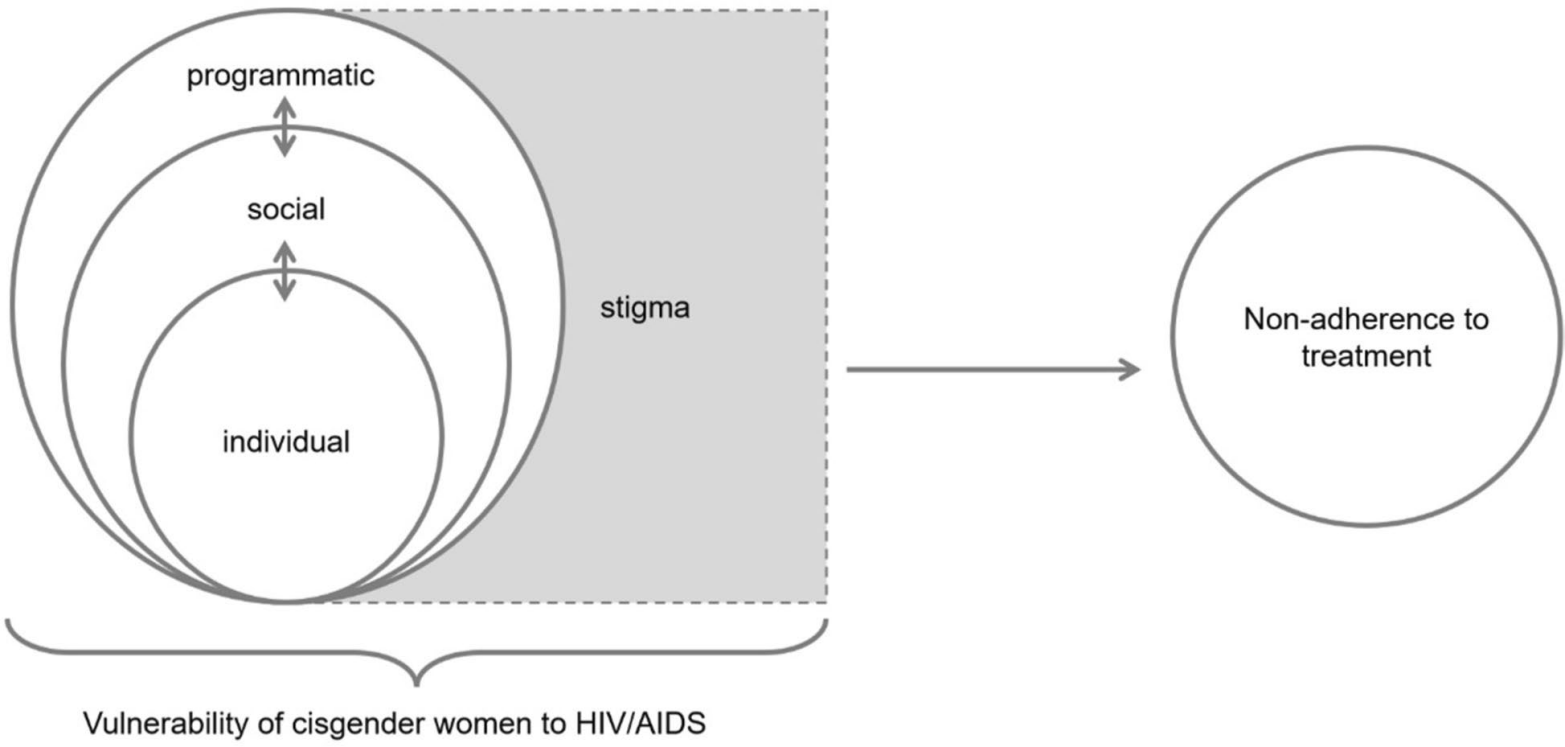
Conceptual model of vulnerability and non-adherence to treatment derived from a scoping review on vulnerability and non-adherence to treatment in cisgender women living with HIV/AIDS (2000–2024). Source: elaboration constructed by the authors

Figure 2 illustrates the proposed model, highlighting how individual, social, and programmatic vulnerabilities interact and reinforce each other, ultimately affecting adherence. Stigma, although classified under the social dimension, is represented as a transversal element permeating all levels of vulnerability.

The model illustrates how individual (e.g., depression/anxiety, substance use, side effects, caregiving burden), social (e.g., anticipated/enacted stigma, gender-based violence, poverty/food insecurity, disclosure dynamics, social support), and programmatic vulnerabilities (e.g., clinic accessibility/transport, waiting times, continuity of care, postpartum follow-up, patient–provider communication) interact across proximal, intermediate, and distal levels to influence adherence and discontinuation. Stigma is represented as a transversal mechanism permeating all dimensions.

The intertwining of these dimensions reveals that many factors are common to two or more categories, adding layers of complexity to their interactions. The combined effects of these vulnerabilities may negatively impact both adherence behaviors and monitoring indicators. Not only do these barriers differ from those faced by men, as it also varies among women, considering their individual and collective diversities. The proposed model thus suggests that analyses of barriers to treatment adherence among WLWHA must account for the distinct dimensions of vulnerability, their interactions, and their effects on treatment outcomes and monitoring.

## Discussion

The findings of this review indicate that adherence to antiretroviral therapy among WLWHA is shaped by a complex interplay of vulnerabilities extending beyond individual behavior. Although numerous studies examined adherence-related factors, many did not explicitly employ the term “vulnerability.” We interpret this as primarily a terminological and theoretical framing gap, rather than absence of vulnerability-related mechanisms: the reported barriers and facilitators map conceptually onto individual, social, and programmatic dimensions.

A systematic review analyzing gender-based differences in adherence identified female sex as a predictor of non-adherence, referencing vulnerability primarily in terms of gender-related factors affecting treatment [90]. Other studies aligned conceptually with vulnerability frameworks but did not explicitly adopt the terminology.

Similarly, a prior scoping review in Brazil emphasized the interplay of individual, social, economic, and structural conditions influencing adherence, noting that the term “vulnerability” has been inconsistently applied despite its conceptual relevance early in the HIV epidemic [91]. Over time, literature focus has shifted toward risk-oriented and epidemiological interpretations, moving away from broader vulnerability perspectives.

The literature’s emphasis on pregnancy and the postpartum period reflects a predominantly clinical and biomedical focus, particularly regarding vertical transmission and antenatal care. This prioritization often marginalizes broader social and structural factors affecting WLWHA’s lives and treatment adherence [44–80]. Consequently, treatment technologies and biomedical outcomes may overshadow women’s lived experiences, neglecting the influence of power structures and social determinants. In the perspective, Foucault’s theory of biopower provides a lens to understand how medicalized governance reinforces control over women’s bodies, particularly concerning sexuality and reproductive roles [92]. The focus on preventing vertical transmission links women’s health responsibilities to reproductive control. Coitinho Filho and Rinaldi [93] highlight how the moralized discourse of undetectability in pregnant women reinforces medicalized oversight of female bodies.

The present review reinforces the interconnection between individual, social, and programmatic dimensions of vulnerability, illustrating how these dimensions compound one another. Individual vulnerabilities are shaped by social inequalities, which are, in turn, structured through institutional and policy frameworks [94–100].

Among all factors, stigma consistently emerged as the most pervasive determinant, operating at multiple levels—internalized fear (individual), discrimination and violence (social), and barriers to healthcare services (programmatic). It exacerbates the vulnerability of WLWHA by undermining both social and institutional relationships. Though typically classified as part of the social dimension [12], it is evident that stigma transcends all levels, making it one of the most powerful forces disrupting adherence. Addressing stigma, alongside gender-based inequalities and structural barriers, is thus central to improving adherence. Overall, the interrelation of vulnerabilities underscores the need for comprehensive approaches that integrate biomedical, social, and structural perspectives, supporting evidence from other HIV-affected populations [101].

While vulnerability provides the primary analytic framework of this review, syndemic theory offers complementary insight into the synergistic nature of co-occurring challenges, helping to interpret how mental health conditions, violence, substance use, and socioeconomic deprivation interact to intensify barriers to sustained antiretroviral treatment adherence [102].

Strengthening adherence requires moving beyond purely biomedical paradigms toward strategies that prioritize social justice, human rights, and structural change. Addressing stigma, along with broader gender-based inequalities and structural barriers, is therefore central to improving adherence. Despite decades of HIV programs focused on women, persistent disparities underscore the need for more integrated responses that value women’s autonomy, recognize their autonomy, and promote equitable access to care. Strengthening adherence in this population requires moving beyond biomedical paradigms toward comprehensive strategies that incorporate social justice, human rights, and structural change.

### Limitations

This scoping review has limitations. First, definitions and measurements of ART adherence/non-adherence varied substantially across studies (e.g., self-report, pharmacy refill, clinical indicators), limiting direct comparability across contexts. Second, consistent with scoping review guidance, we did not conduct critical appraisal of individual sources; therefore, the mapped evidence reflects the breadth of available literature rather than strength of causal inference. Third, despite efforts to include gray literature, our approach may not have captured all non-indexed sources and may be influenced by publication bias. Fourth, language capacity and translation support may have introduced residual language bias, particularly for full texts beyond the team’s primary languages. Finally, the relatively high proportion of pregnancy/postpartum-focused studies may shape the prominence of these contexts in the synthesis, and findings may not fully represent vulnerabilities outside reproductive care pathways.

## Conclusions

This scoping review demonstrates that barriers to adherence to antiretroviral therapy among cisgender WLWHA is shaped by interconnected vulnerability contexts spanning individual, social, and programmatic dimensions. Adherence challenges are not limited to personal responsibility but emerge from the intersection of mental health conditions, socioeconomic inequalities, gender-based violence, stigma, and structural barriers within health systems.

Recognizing these overlapping vulnerabilities highlights the need for comprehensive strategies that integrate biomedical, social, and structural perspectives. Interventions must address clinical factors while simultaneously confronting gender inequalities, racial and socioeconomic disparities, and institutional practices that reinforce exclusion.

Importantly, women’s autonomy must be acknowledged many navigate treatment while managing family responsibilities, negotiating disclosure, and mobilizing social support. Policies and programs should build on this autonomy while actively reducing systemic inequities. Effectively addressing HIV among women requires moving beyond risk-focused and biomedical approaches toward rights-based, equity-driven responses. Reducing stigma, ensuring equitable access to care, and promoting social justice are essential to enhance adherence and ultimately improve the health and well-being of WLWHA. While several recommendations—such as strengthening continuity of care, reducing structural barriers to access, and implementing stigma and violence-informed services—emerged across multiple regions, some policy levers are context-dependent and should be adapted to local health system organization, resource availability, and sociocultural conditions.

## Supplementary Information

Supplementary Material 1.

Supplementary Material 2.

## Data Availability

The datasets generated and/or analyzed during the current study, based on search strategies, are available on: Scopus [103]; Web of Science [104]; SciElo [105]; DLTD [106]; PubMed [107].

## Abbreviations

AIDS: Acquired Immunodeficiency Syndrome
ART: Antiretroviral Therapy
BAD: Bipolar Affective Disorder
DLTD: Digital Library of Theses and Dissertations
HIV: Human Immunodeficiency Virus
IPV: Intimate Partner Violence
JBI: Joanna Briggs Institute
MeSH: Medical Subject Headings
OSF: Open Science Framework
PCC: Population-Concept-Context
PLWHA: People Living with HIV/AIDS
PRISMA-ScR: Preferred Reporting Items for Systematic Reviews and Meta-Analyses - Scoping Review Extension
PTSD: Post-Traumatic Stress Disorder
SDH: Social Determinants of Health
SciELO: Scientific Electronic Library Online
WLWHA: Women Living with HIV/AIDS
